# Management of *Pseudomonas aeruginosa* Bloodstream Infection and Impact on Health Outcomes

**DOI:** 10.1017/ash.2021.41

**Published:** 2021-07-29

**Authors:** Swetha Ramanathan, Margaret Fitzpatrick, Fritzie Albarilo, Katie Suda, Linda Poggensee, Amanda Vivo, Martin Evans, Makoto Jones, Nasia Safdar, Geneva Wilson, Charlesnika Evans

## Abstract

**Background:** Gram-negative bacteria cause a variety of hospital-associated infections (HAIs). Of concern is *Pseudomonas aeruginosa*, which is a leading cause of HAIs. Early and adequate therapy of *P. aeruginosa* blood stream infection (BSI) is associated with decreased mortality. Additionally, infectious disease consultation has also shown to improve health outcomes, streamline care, and decrease costs. Therefore, the goal of this study was to describe treatment of *P. aeruginosa* BSI and impact of infectious disease consultations on health outcomes. **Methods:** In this retrospective cohort study, we analyzed national VA medical, encounter, pharmacy, microbiology, and laboratory data from January 1, 2012 to December 31, 2018. The cohort included all hospitalized adult veterans (aged ≥18 years) who had a positive blood culture for *P. aeruginosa*. Only the first *P. aeruginosa* blood culture per patient was included, and duplicate cultures within 30 days were removed. Treatment was identified within −2 to +5 days of the culture date. Multidrug-resistant (MDR) cultures were identified based on resistance to at least 1 agent in at least 3 or more antimicrobial categories tested. Multivariable logistic regression models were fit to assess infectious disease consultations and adequate treatment on in-hospital mortality and 30-day mortality. **Results:** In total, 3,256 patients had a BSI with *P. aeruginosa*, of which 386 (11.5%) were MDR. Most of these patients were male (97.5%), >65 years of age (70.9%), and non-Hispanic white (63.8%). Also, 784 patients (23.3%) died during hospitalization and 870 (25.8%) died within 30 days of their culture. In multivariable regression models, infectious disease consultations were associated with decreased odds of in-hospital mortality (odds ratio [OR], 0.64; 95% confidence interval [CI], 0.53–0.77) and 30-day mortality (OR, 0.56, 95% CI, 0.48–0.67) even after adjusting for age, race, care setting, Charlson score, and prior healthcare exposures. Furthermore, inadequate definitive treatment was associated with increased odds of in-hospital mortality (OR, 2.77; 95% CI, 1.35–5.69) and 30-day mortality (OR, 2.37; 95% CI, 1.18–4.79), even after adjusting for age, Charlson score, care setting, and prior healthcare exposures. In addition, carbapenem treatment was associated with increased odds of in-hospital mortality (OR, 1.38; 95% CI, 1.12–1.70) and 30-day mortality (OR, 1.49; 95% CI, 1.22–1.81), whereas fluoroquinolone treatment was associated with lower odds of in-hospital mortality (OR, 0.49; 95% CI, 0.41–0.59) and 30-day mortality (OR, 0.60; 95% CI, 0.50–0.71). Finally, extended-spectrum cephalosporin was also associated with lower odds of in-hospital mortality (OR, 0.82; 95% CI, 0.68–0.98). **Conclusions:** Use of infectious disease consultations and any adequate definitive treatment for those with *P. aeruginosa* BSI lowered odds of in-hospital and 30-day mortality. Early consultation with infectious disease physicians regarding adequate treatment has direct positive impact on clinical outcomes for patients with *P. aeruginosa* BSI.

**Funding:** No

**Disclosures:** None

